# Reviews of Dental Literature

**Published:** 1901-04

**Authors:** 


### Reviews of Dental Literature.

ACTINOMYCOSIS.—MM. Berard and Nicholas have proved that the spores of this parasite can preserve their vitality more than six years. They are killed by fourteen minutes' exposure to dry heat at 80° C. Sunlight kills them in fourteen and a half hours when in a liquid; when dry they are unaffected.—*L'Odontologie.*

A TREATMENT OF PULPLESS TEETH, a paper read at the International Congress. By M. L. Quintin, of Brussels.

The writer reports seven thousand cases treated in the following manner, with but one failure.

Where there is no pericementitis he washes the canals with hydrogen peroxide, twelve volumes, dries and seals the apex with a paste made of iodoform and five per cent. formalin. Where there is pericementitis he washes with peroxide and leaves the tooth open, save for cotton loosely packed, for a day. At the next sitting, after washing again with peroxide, he pumps a drop of forty per cent. formalin into the canals, packs the canals with filaments of earth wet with five per cent. formalin, and leaves till another sitting, at which time he dries and fills with the iodoform and formalin paste.

It was in this class that he met with his one failure in seven thousand (quite a notable showing), in the case of a chlorotic young girl who demanded the immediate extraction of the offending tooth, which proved to have a sac twice the size of the crown of the tooth.

When there is pericementitis, and a fistulous opening exists, he washes, dries, and fills with paste at once, the fistula healing after three or four days. At this point he remarks that in the case of certain teeth, the incisors particularly, it is necessary to proceed with some caution when the fistula is of long standing. In such cases the apical foramen is liable to be larger than is common, and it is necessary to guard against the danger of pushing the paste through into direct contact, in any considerable quantity, with the pericementum.

As the writer says in his paper, there is nothing especially new in this treatment, but the success of the method, one failure in seven thousand cases, is greater than most advocates of other systems of root-filling can claim.

Doubtless among the many antiseptic powders at our command a substitute could be found for the objectionable iodoform.—*L'Odontologie.*

Syphilitic Localosis Alveolaris (Pyorrhœa Alveolaris). By E. Lennox Curtis, M.D.

Dr. Curtis believes that many, or, it would seem, most, cases of pyorrhœa are due to a syphilitic taint. Where the white patches that one sees in the mucous membrane of the cheeks of some patients, which he well terms "egg-skin eschar," are present, he feels especially sure of his diagnosis. But to confirm it he has examinations made of blood fresh from the patient. "The examination of more than one hundred cases revealed strong evidences of syphilis, and in every instance when the egg-skin eschar was present the blood showed unmistakable proofs of the taint; in fact, in every case where the blood showed this the egg-skin eschar was present." As proof of his theory Dr. Curtis cites the fact that such cases get well and remain so when placed on antisyphilitic treatment.—*Dental Cosmos* for February.

The Best Filling-Material for the Temporary Teeth. By John J. Bushe, D.D.S.

For the temporary teeth alone Dr. Bushe recommends copper amalgam, for the following reasons:

"It prevents recurrence or extension of decay, being antiseptic and non-shrinkable. It is so plastic that its manipulation is a delight. It is so slow setting that no haste need be made in the operation, although its easy working makes it possible to put in a filling more quickly than any other material. It may be placed in close proximity to the pulp, being non-irritating, and can be inserted without making pressure thereon. It is not affected by the presence of saliva, either in the cavity or on the unfinished filling, as are all the other plastics; no special pains to have perfect dryness is necessary. The filling is more easily and quickly finished than that made from any other substance."

To these well-founded statements Dr. Bushe adds that in ten years' experience he has never found any wasting of the amalgam in temporary teeth.

GOLD BLINDNESS, OR RETINAL ASTHENOPIA, AND ITS TREATMENT. By L. Webster Fox, A.M., M.D.

Yellow rays stimulate the retina and after a time cause it to become exhausted (retinal asthenopia) to the degree that in the eyes of those most readily affected a blind spot temporarily exists. From this it happens that an operator sometimes finds the outline of the cavity he is filling growing indistinct, or even disappearing. Those suffering from hypermetropia with some astigmatism, or from muscular insufficiency, are most inclined to be thus affected.

The treatment is to improve the general health, and to wear glasses calculated to correct visual defects, the glasses to be of a slightly violet color, to overcome the effect of the yellow rays.

